# Linking Motoneuron PIC Location to Motor Function in Closed-Loop Motor Unit System Including Afferent Feedback: A Computational Investigation

**DOI:** 10.1523/ENEURO.0014-20.2020

**Published:** 2020-04-27

**Authors:** Hojeong Kim

**Affiliations:** Division of Biotechnology, Convergence Research Institute, DGIST, Daegu 42988, Korea

**Keywords:** dendrites, L-type Cav1.3 channel, motoneuron, motor unit, muscle spindle, persistent inward current

## Abstract

The goal of this study is to investigate how the activation location of persistent inward current (PIC) over motoneuron dendrites is linked to motor output in the closed-loop motor unit. Here, a physiologically realistic model of a motor unit including afferent inputs from muscle spindles was comprehensively analyzed under intracellular stimulation at the soma and synaptic inputs over the dendrites during isometric contractions over a full physiological range of muscle lengths. The motor output of the motor unit model was operationally assessed by evaluating the rate of force development, the degree of force potentiation and the capability of self-sustaining force production. Simulations of the model motor unit demonstrated a tendency for a faster rate of force development, a greater degree of force potentiation, and greater capacity for self-sustaining force production under both somatic and dendritic stimulation of the motoneuron as the PIC channels were positioned farther from the soma along the path of motoneuron dendrites. Interestingly, these effects of PIC activation location on force generation significantly differed among different states of muscle length. The rate of force development and the degree of force potentiation were systematically modulated by the variation of PIC channel location for shorter-than-optimal muscles but not for optimal and longer-than-optimal muscles. Similarly, the warm-up behavior of the motor unit depended on the interplay between PIC channel location and muscle length variation. These results suggest that the location of PIC activation over motoneuron dendrites may be distinctively reflected in the motor performance during shortening muscle contractions.

## Significance Statement

How may the location of persistent inward current (PIC)-generating Ca_v_1.3 channels over spinal motoneuron dendrites affect the force production of the motor unit? To systematically investigate this fundamental issue, a physiologically realistic model of closed-loop motor unit including muscle spindle feedback is built. The computational analysis of model motor unit hierarchically reveals the functional link of the PIC channel location in motoneuron with the input-output properties of closed-loop motor unit under isometric contraction at various muscle lengths. The present study reports that the motor performance during shortening muscle contractions may distinctively reflect the location of PIC activation over motoneuron dendrites. These results would provide useful insights into spinal mechanisms and motor unit function in control of movement.

## Introduction

A single spinal motoneuron makes direct contacts with a bundle of muscle fibers, forming a motor unit, which is known as the smallest functional unit underlying all motor activities. The force output generated by the motor unit has been shown to be determined not only by neural signals conducted to the muscle fibers from the motoneuron but also by the interaction of muscle activation dynamics with muscle length variations during motor activities (Sandercock and Heckman, 1997; Heckman and Enoka, 2012).

Spinal motoneurons have the capability to generate nonlinear firing behavior (Hounsgaard and Kiehn, 1989; Lee and Heckman, 1996; Carlin et al., 2000; Bennett et al., 2001; Gorassini et al., 2002a). In the presence of monoamines such as serotonin or norepinephrine, the nonlinearity in firing patterns has been characterized as a counterclockwise hysteretic relationship between firing rate and stimulation intensity during slowly ascending and descending current injection at the soma or synaptic inputs over the dendrites (Powers et al., 2012). This behavior is featured by acceleration of the firing rate at current intensities higher than the current threshold for firing initiation in the ascending phase (firing rate potentiation) and persistence of firing below the current threshold for firing initiation in the descending phase (self-sustaining firing; Lee and Heckman, 1998a,b).

The nonlinear firing of motoneurons has been shown to be mediated primarily by persistent inward current (PIC)-generating L-type Ca_v_1.3 channels clustered over dendritic trees (Hounsgaard and Kiehn, 1993; Carlin et al., 2000; Lee and Heckman, 2000). The dynamics of PIC activation strongly depends on the location of L-type Ca_v_1.3 channels over motoneuron dendrites (Elbasiouny et al., 2005; Bui et al., 2006; Carlin et al., 2009). This location dependence of PIC activation is determined by both the morphologic and passive electrical properties of the dendrites (Rall and Rinzel, 1973; Zador et al., 1995; Magee, 2000). Anatomical and electrophysiological studies have shown the significant variation in structural and electrical characteristics across spinal motoneuron pool (Cullheim et al., 1987; Fleshman et al., 1988; Zengel et al., 1985). One study suggested more proximal location of PIC channels to the soma in small motoneurons (Grande et al., 2007). The other study demonstrated more distal location of PIC channels to the soma in low-threshold motoneurons to match the electrical properties observed in experiments (Kim, 2017a). Optical imaging studies combining immunohistochemistry for PIC channels have reported the disperse distribution of PIC channel density over the soma and dendrites (Ballou et al., 2006; Sukiasyan et al., 2009). Furthermore, the location effect on PIC activation may vary with changes in the morphologic and electrical factors of dendrites with pathologic damage (Elbasiouny et al., 2010; Šišková et al., 2014) or in space environment (Parihar et al., 2015). Thus, it is natural to assume that the location of PIC activation can vary over motoneuron dendrites even for the same type of spinal motoneurons. Yet, little is known on whether and how the variability of PIC activation location may influence the motor output during motor activities.

The functional role of PIC activation location in motor control may not be fully inferred from the discharge patterns of the motoneuron. It is well known that muscle force is nonlinearly related with excitation frequency (Mannard and Stein, 1973) and muscle length (Scott et al., 1996). Recently the level of muscle activation has been reported to be even more degraded, particularly under twitch and subtetanic (i.e., <20 Hz) contractions with muscles shortened below the optimal length at which the muscle produces the maximum force during isometric condition under full excitation (Perreault et al., 2003; Kim et al., 2015). Furthermore, the afferent inputs from muscle spindle to spinal motoneurons under variation in muscle length may further increase the complexity in the interactions between PIC activation location, motoneuron discharge and motor function in the motor unit (Lennerstrand, 1968).

Here, we comprehensively investigated how the PIC location and muscle length interplay on motor performance in the closed-loop motor unit system with afferent feedback from muscle spindle. The influence of muscle afferent feedback was first analyzed extending an open-loop motor unit model to include the physiological distribution of muscle spindle afferent inputs over the motoneuron dendrites. Then, the physiological input-output properties of the closed-loop motor unit model were assessed while varying the PIC location along the paths of motoneuron dendrites and the muscle length spanning the full physiological range, measuring three characteristic indices representing the capability of self-sustaining force generation, the rate of force development and the degree of force potentiation.

## Materials and Methods

### Modeling closed-loop motor unit system

**Figure 1.**
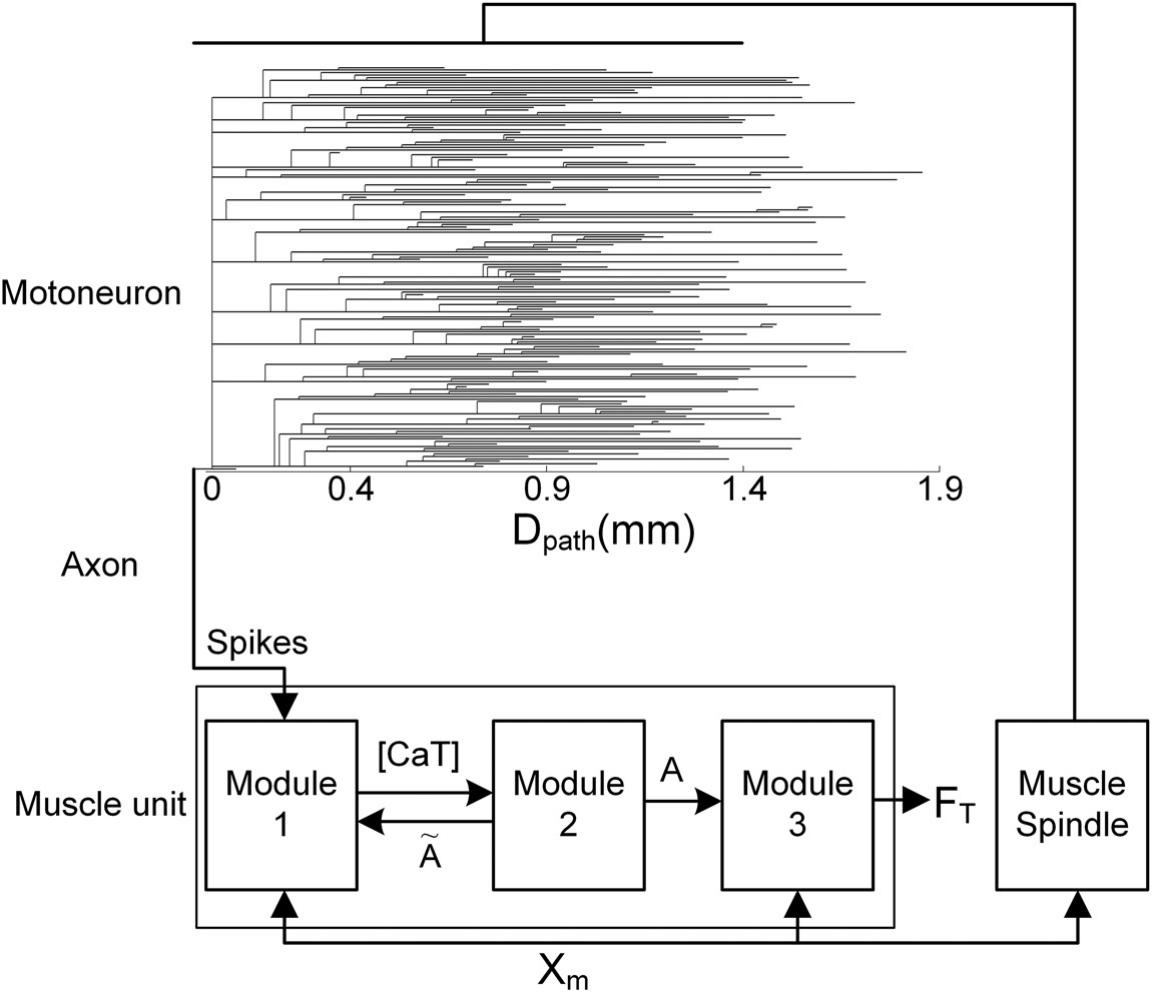
Schematic diagram of the model motor unit including muscle afferents. Top, Dendrogram of the motoneuron and a uniform distribution of afferent inputs from the muscle spindle over the dendrites up to a path length (i.e., D_path_) of 1.4 mm from the cell body. Spikes indicate the voltage output generated at the soma of the motoneuron. Bottom, Muscle unit model comprising three modules (module 1, module 2, and module 3). [CaT], *A*, F_T_, and X_m_ indicate the concentration of calcium bound to troponin, the muscle activation level, the force output, and the muscle length, respectively.

The model closed-loop motor unit comprises three components: a motoneuron, muscle fibers and a muscle spindle (Fig. 1). The modeling procedures, model equations and parameter values for the motoneuron and muscle fibers of the motor unit model were fully presented in a previous study (Kim, 2017b). In the present study, the efferent and afferent nerves were assumed to perfectly transfer the action potentials from the motoneuron and the muscle spindle and were modeled as a single parameter representing the conduction delay (i.e., 10 ms) over the nerve.

#### Motoneuron

The morphologic data for adult cat α-motoneuron (i.e., vemoto6) were downloaded from a public database (www.neuromorpho.org), reconstructed in the NEURON software environment (Hines and Carnevale, 1997) and corrected to reflect the soma geometry as reported in the previous study (cell 43/5; Cullheim et al., 1987). The axonal hillock and initial segment were modeled as a 20-μm-long cable tapering with a 13-μm initial and 3.3-μm end diameter and a 30-μm-long cable with a constant diameter of 3.3 μm (Kellerth et al., 1979) and attached to the soma of the motoneuron model. The passive membrane properties (i.e., nonuniform-specific membrane resistivity at the soma and the dendrites and uniform-specific membrane capacitance and axial resistivity for all compartments) were directly adopted from the literature (Fleshman et al., 1988). The active membrane mechanisms responsible for producing action potentials included fast sodium, delayed rectifier potassium, N-type calcium, Ca-dependent potassium, and persistent sodium currents over the soma and fast sodium, delayed rectifier potassium and persistent sodium currents over the axonal hillock and the initial segment. The dynamics of intracellular calcium concentration in the soma were adjusted so that the speed coupling between the afterhyperpolarization (AHP) duration of the motoneuron action potential and the duration of the twitch response by the muscle fibers was matched during isometric contraction at the optimal muscle length, as reported experimentally for slow motor units (∼250 ms; Iaizzo and Poppele, 1990; Bakels and Kernell, 1993). PICs responsible for nonlinear (or bistable) firing behavior were produced by including L-type Ca_v_1.3 channels over the dendrites. Sodium channels (i.e., Na_v_1.1 and 1.6) contributing to PICs were not considered since they have been shown to be responsible mainly for spiking initiation (Harvey et al., 2006).

#### Muscle unit

Force was generated using the previous muscle model, which accurately replicated force production of the adult cat soleus over a full physiological range of input conditions for stimulation rate (i.e., 1–100 Hz) and muscle length (i.e., −16 to 0 mm) during isometric, isokinetic and dynamically varying length conditions (Kim et al., 2015). Briefly, the model consists of three modules, each of which represents signal transformations observed experimentally during muscle contraction. The first module (i.e., module 1) transforms action potentials from the motoneuron to the concentration dynamics of calcium ions in the sarcoplasm. The second module (i.e., module 2) transforms of the calcium concentration into the level of muscle activation reflecting the amount of crossbridge formations. The third module (i.e., module 3) transforms the level of muscle activation to the force based on Hill-type mechanics.

#### Muscle spindle

Synapses of afferent inputs from muscle spindles for sensory feedback were added over the soma and the dendritic areas at a path length <1.4 mm from the soma based on the results of a previous study (Segev et al., 1990). The muscle spindle afferent inputs were placed uniformly and activated synchronously over the motoneuron considering the activation of both group Ia and II afferent inputs during isometric variation in muscle length (Boyd, 1980). The peak conductance (i.e., G_aff_) of the afferent inputs was determined for three muscle lengths (physiologically minimal, optimal and maximal) by matching the experimental data for effective synaptic current (i.e., I_N_) measured at the soma of adult cat motoneurons under voltage clamping at the cell body while slowly increasing the muscle length from the physiologically minimal (i.e., −10 mm) to maximal (i.e., 0 mm) muscle length (Lee et al., 2003). The G_aff_ was set to 0 μS/cm^2^, 9.3 μS/cm^2^, and 19 μS/cm^2^ for I_N_ of 0 nA at −16 mm, 2.5 nA at −8 mm, and 5 nA at 0 mm, respectively. It should be noted that the effects of motor unit contraction on feedback signals from muscle spindle and tendon organ were not involved in the current version of motor unit model assuming the quick disappearance of interaction between motor unit and muscle and tendon mechanoreceptor in slow motor units (Zytnicki et al., 1990; Lafleur et al., 1992).

### Location variation of motoneuron PIC

L-type Ca_v_1.3 channels responsible for producing PICs experimentally observed in spinal motoneurons were distributed over the dendrites based on the hot-spot hypothesis (Bui et al., 2006). That is, the PIC channels were clustered over the limited dendritic areas rather than distributed uniformly over the entire dendritic branches. In this study, the PIC channels were located over all dendritic branches that were separated from the soma by similar path length. The locations of the PIC channels were varied centrifugally from the soma to the tips of dendritic trees. The peak conductance (i.e., G_CaL_) of the L-type Ca_v_1.3 channels was operationally adjusted according to the location of the channels over the dendrites so that the effective calcium current (i.e., 22 nA) approaching the soma was held constant during voltage clamping at the soma (see Table 1 for the values of G_CaL_; Lee and Heckman, 1998a, 1999). It should be noted that low voltage activated L-type calcium channels (i.e., Ca_v_1.3) were used as the primary source for producing the calcium-related PICs underlying bistable firing behavior that have been experimentally observed at the soma in previous studies (Hounsgaard and Kiehn, 1989; Carlin et al., 2000).

**Table 1:** Peak conductance of persistent calcium current (G_CaL_, mS/cm^2^) at various path lengths (D_path_, mm) and corresponding electrotonic lengths (mean ± SDλ) along the dendrites in the model of motor unit

	0.1 mm	0.2 mm	0.3 mm	0.4 mm	0.5 mm	0.6 mm	0.7 mm	0.8 mm	0.9 mm	1.0 mm
D_path_	0.04 ± 0.01λ	0.07 ± 0.02λ	0.11 ± 0.03λ	0.16 ± 0.04λ	0.21 ± 0.04λ	0.26 ± 0.04λ	0.3 ± 0.05λ	0.36 ± 0.05λ	0.41 ± 0.06λ	0.46 ± 0.07λ
G_CaL_	1.57	1.14	1.21	1.25	1.28	1.37	1.39	1.95	2.8	4.1

### Length variation of muscle unit

The muscle length was varied under isometric conditions at three different states: physiologically minimal (i.e., −16 mm), optimal (i.e., −8 mm), and maximal (i.e., 0 mm). The physiological range of muscle length was determined based on experimental measurements of length variation in cat soleus muscles during slow walking (Goslow et al., 1973). The variation in muscle length influenced both the muscle activation and the synaptic drive to the motoneuron dendrites. The level of muscle activation was modulated by considering the concentration of calcium bound to troponin (i.e., CaT) in the sarcoplasm as a function of muscle length. The synaptic drive to the motoneuron was augmented by the addition of afferent inputs from the muscle spindle, which depended on the muscle length.

### Stimulation protocols

To investigate the input-output properties of the model closed-loop motor unit, the somatic and dendritic stimulation protocol used in previous studies on motoneuron physiology was applied at the soma and the dendrites of the motoneuron. For the somatic stimulation, slowly ascending and descending intracellular current (i.e., I_S_) was injected at the soma with a peak of 20 nA at 5 s (Lee and Heckman, 1998b). For the dendritic stimulation, the excitatory synapses were additionally placed in the dendritic regions over which the PIC channels were located. The peak conductance (i.e., G_syn_) of excitatory synaptic inputs over the dendrites slowly increased and then decreased in a triangular form with a peak of 1.2 mS/cm^2^ at 10 s (Powers and Heckman, 2017). For direct comparison with the current input-force output relationship under intracellular stimulation at the soma, the effective synaptic current (i.e., I_N_) was calculated at the soma under voltage clamping while varying the triangular G_syn_ over the passive dendrites (Heckman and Binder, 1988). The peak value of G_syn_ was selected for production of the maximum I_N_ of 16 nA that represents a synaptic activation level in the dendrites that is ∼350% of that produced by Ia afferents alone (Elbasiouny et al., 2006). The input-output relationship under the synaptic stimulation condition was represented by the relationship between I_N_ and force.

The warm-up behavior of the motoneuron was induced by repeatedly stimulating over the soma and the dendrites as shown in the experimental studies (Bennett et al., 1998). For the somatic stimulation, triangular current stimulation (−5 nA at start and 10 nA at peak for a 2-s period) was repeated at the soma of the motoneuron over 8 s. The interactivation interval (∼1 s) was determined based on the human study showing the short interaction intervals (1–2 s) are more effective to induce the warm-up phenomenon (Gorassini et al., 2002b). For the dendritic stimulation, sinusoidal tendon movement from physiologically minimal and maximal muscle length over a 2-s period was simulated by varying the maximum conductance (G_aff_) of afferent synapses over the motoneuron dendrites in a sinusoidal form with the period of 2 s. The minimum (0 μS/cm^2^) and maximum value (19 μS/cm^2^) of sinusoidal variation in G_aff_ were set to those estimated at the physiologically minimal (X_m_ = −16 mm) to maximal muscle length (X_m_ = 0 mm), respectively. The step current (−1 nA at start and −0.5 nA at transition after 4 s) was applied at the soma while the sinusoidal variation of G_aff_ was repeated for 16 s as in the previous study. It is noted that during the dendritic stimulation all voltage-gated ion channels over the soma, axonal hillock and initial segment were blocked to simulate the firing inactivation with intracellular administration of QX-314.

### Input-output analysis

**Figure 2.**
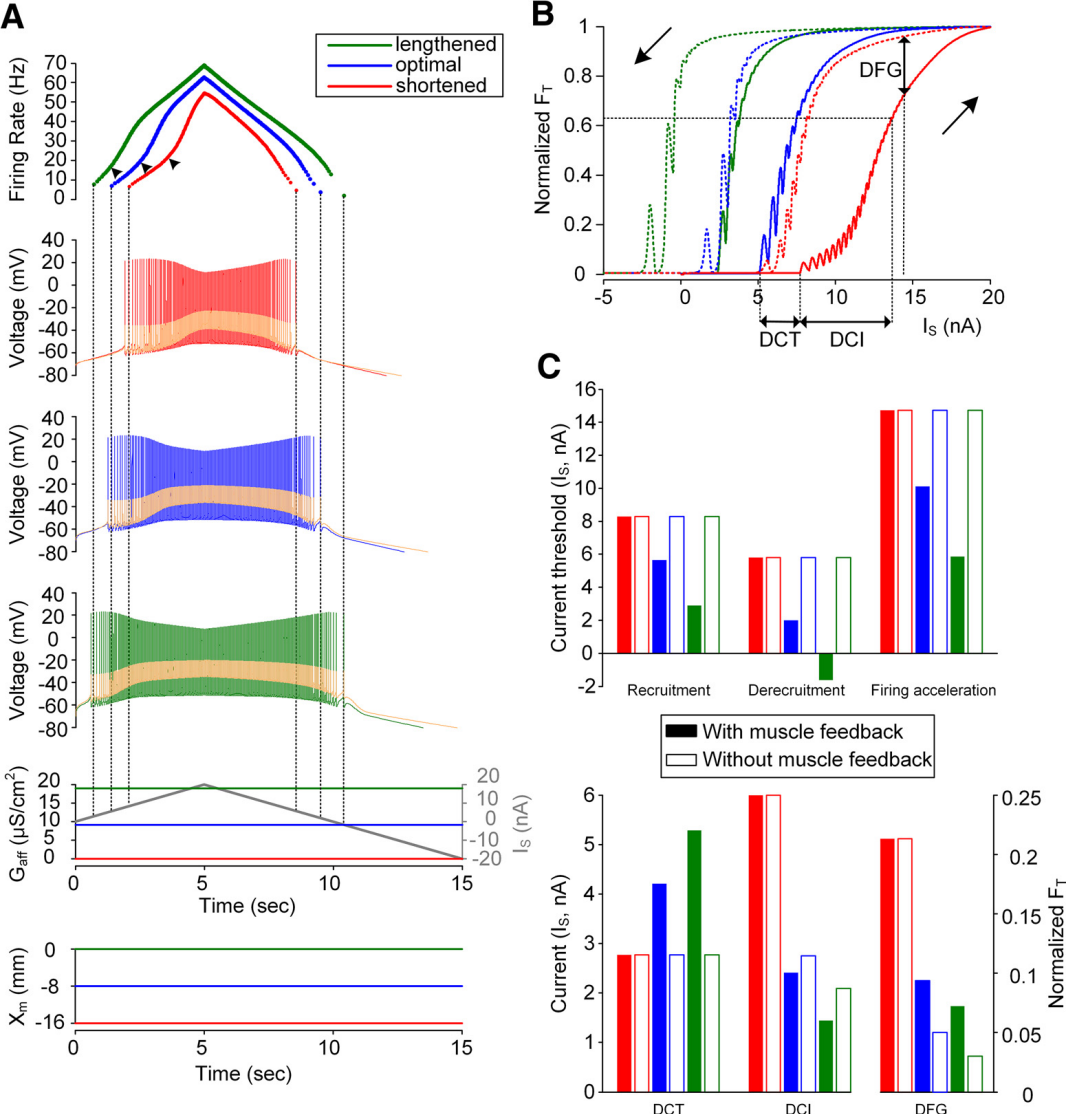
Influence of muscle spindle feedback on somatic input-force output relationship of a model motor unit. ***A***, Instantaneous firing rate of the motoneuron with PIC channels at a D_path_ of 0.6 mm (top) in response to the triangular current injected at the soma of the motoneuron (I_S_) and the afferent inputs from the muscle spindle (G_aff_; bottom) during isometric contractions at shortened (X_m_ = −16 mm), optimal (X_m_ = −8 mm), and lengthened muscle length (X_m_ = 0 mm). Vertical dotted lines indicate the recruitment and derecruitment and black arrowheads indicate the onset of firing acceleration induced by full PIC activation. ***B***, The relationship between the current (I_S_) and force output (F_T_) normalized with peak force for each muscle length. The solid and dotted lines indicate the forces produced during the ascending and descending stimulation phases, respectively. The two black arrows indicate the direction of the stimulation phase. The DCT, DCI, and DFG values are the characteristic indices representing the capability of self-sustaining force production, the rate of force development and the degree of force potentiation by PIC activation. ***C***, Bar graph for the current threshold for recruitment, derecruitment and PIC activation in the motoneuron (upper panel) and the characteristic indexes for motor output (bottom panel). Filled and empty bars represent the simulation data with and without muscle spindle feedback to the motoneuron.

**Figure 3.**
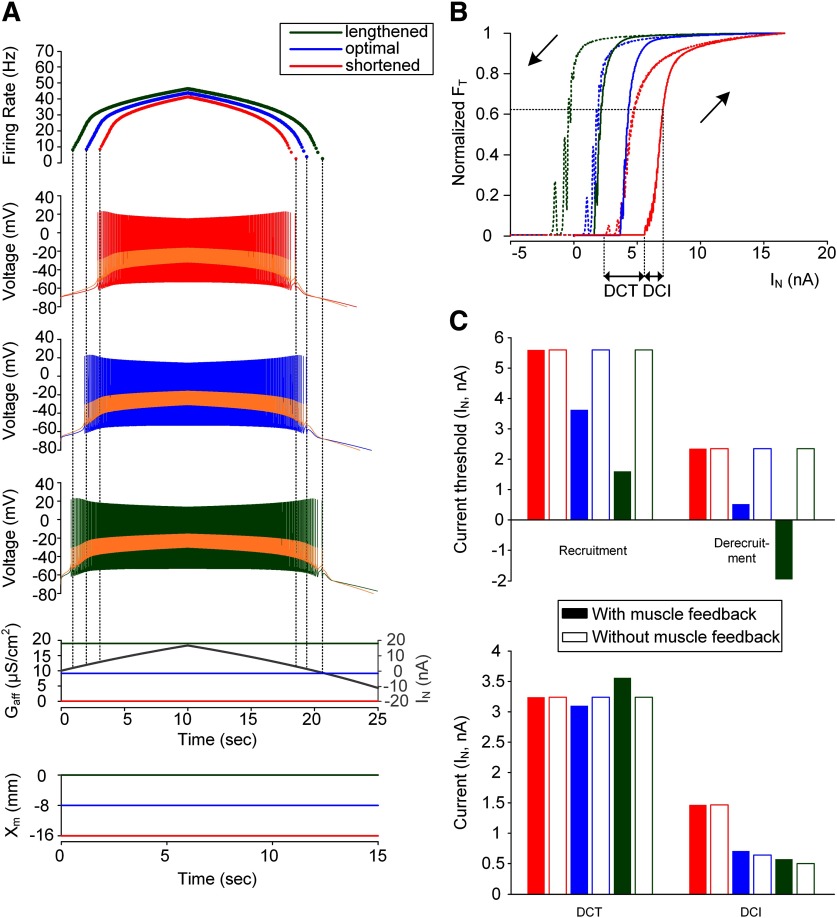
Influence of muscle spindle feedback on dendritic input-force output relationship of a model motor unit. ***A***, Instantaneous firing rate of the motoneuron with PIC channels at a D_path_ of 0.6 mm (top) in response to the effective synaptic current approaching the soma during the triangular variation in the peak conductance of the synaptic inputs over the passive dendrites of the motoneuron (I_N_) and the afferent inputs from the muscle spindle (G_aff_; bottom) during isometric contractions at shortened (X_m_ = −16 mm), optimal (X_m_ = −8 mm), and lengthened muscle length (X_m_ = 0 mm). Vertical dotted lines indicate the recruitment and derecruitment. ***B***, The relationship between the effective synaptic input (I_N_) and force output (F_T_) normalized with peak force for each muscle length. The solid and dotted lines indicate the force produced during the ascending and descending stimulation phases, respectively. The two black arrows indicate the direction of the stimulation phase. The DCT and DCI values are the characteristic indices representing the capability of self-sustaining force production and the rate of force development. The DFG value is not indicated due to the simultaneous onset of firing and PIC under the synaptic inputs to the dendrites. ***C***, Bar graph for the current threshold for recruitment and derecruitment in the motoneuron (upper panel) and the characteristic indexes for motor output (bottom panel). The current threshold for PIC activation and the DFG were not included due to the full activation of PIC at the onset of firing during the ascending stimulation phase for all muscle lengths. Filled and empty bars represent the simulation data with and without muscle spindle feedback to the motoneuron.

The motor output of the model closed-loop motor unit was characterized using three characteristic indices (Figs. 2*B*, 3*B* for graphical representations). The difference in current threshold (DCT) for force production between the ascending and descending stimulation phases of the motoneuron was calculated to evaluate the capability of self-sustaining force generation induced by PIC activation at the motoneuron dendrites. A larger DCT indicates a broader range of current intensity over which the motor unit can produce force in the descending phase below the current threshold for force initiation of the ascending phase. The difference in current intensity (DCI) was calculated as the difference in the current threshold required for force initiation from the current intensity at which the force approaches 63% of the peak force. A larger 1/DCI represents a faster rate of force development during the ascending phase of stimulation at the motoneuron. The difference in force generation (DFG) between the ascending and descending stimulation phases was measured at the current intensity at which the firing rate of the motoneuron started to accelerate due to the initiation of full PIC activation in the motoneuron dendrites on the ascending phase. The current intensity for DFG evaluation was determined from the frequency-current relations of the motoneuron. The DFG was calculated only for motoneurons that displayed the ability to transition between two stable firing states with different frequencies (i.e., bistability). Otherwise, the value of DFG was set to be zero. Alarger DFG indicates greater force potentiation by PIC activation. For the purpose of comparison between different conditions of PIC activation location and muscle length, the properties of motoneuron output and force generation were displayed on a bar chart. It should be noted that any random effects (i.e., background synaptic activity) were not included in the present study.

### Simulations

The input-output analysis of the closed-loop motor unit system including muscle afferents was conducted for two situations that have been considered experimentally: (1) the combination of sensory and intracellular stimulation at the soma of the motoneuron during isometric muscle contractions at various lengths and (2) the combination of sensory and central excitation to the dendrites of the motoneuron during isometric muscle contractions at various lengths. All simulations were performed under the NEURON software environment (version 6.1.1) using the built-in integration method (CNEXP) with a fixed time step (0.025 ms) in a desktop computer operated by 64-bit Microsoft Windows 10. The stability and accuracy of simulations were ensured while varying the parameter values over a wide range.

### Code accessibility

The computer codes of models and simulations developed for this study were presented as Extended Data 1 and are also available at the public repository of ModelDB (https://senselab.med.yale.edu/modeldb/).

10.1523/ENEURO.0014-20.2020.ed1 Extended Data 1 Computer codes for the closed-loop motor unit model and simulations. Download Extended Data 1, ZIP file.

## Results

The input-output properties of the closed-loop motor unit model were investigated under somatic and dendritic stimulation conditions in the motoneuron during isometric contractions at physiologically minimal, optimal and maximal states of muscle length (X_m_ = −16, −8, and 0 mm). First, the influence of muscle afferent inputs on the input-output relationship of the motor unit was characterized with PIC channels clustered at ∼0.6 mm along the path of the motoneuron dendrites from the soma [i.e., a path length (D_path_) of ∼0.6 mm]. Second, the interplay of two factors (D_path_ and X_m_) on force output was compared under the following three representative D_path_ conditions: proximal D_path_ (0.1–0.3 mm with the mean of 0.2 mm), intermediate D_path_ (0.5–0.7 mm with the mean of 0.6 mm), and distal D_path_ (0.9–1.1 mm with the mean of 1.0 mm) for locations of PIC channels on the motoneuron (Elbasiouny et al., 2005). Third, the overall shape of the functional link between the two-factor interaction and the input-output properties of the motor unit was summarized over a wide range of electrotonic length over the motoneuron dendrites. Finally, the warm-up behavior was investigated under variation in the two factors.

### Influence of muscle spindle feedback on motor output under somatic stimulation

Figure 2 shows how alterations in muscle length influence the activation of motoneuron PIC channels located at the D_path_ of ∼0.6 mm and the resulting muscle force under intracellular stimulation at the soma. As the X_m_ increased from the physiologically minimal (i.e., −16 mm) to maximal (i.e., 0 mm) length, the current values for motoneuron recruitment (vertical dotted lines), firing acceleration induced by PIC activation (arrowheads) and derecruitment (vertical dotted lines) during the triangular stimulation (I_S_) were reduced by 32%, 30% and 66%, respectively, for the optimal X_m_ (blue) and by 65%, 62%, and 128% for the maximal X_m_ (green) compared with the minimal X_m_ case (red; Fig. 2*A*,*C*, top panel). This result was due to the increase in excitatory afferent inputs (G_aff_) from the muscle spindle as the X_m_ increased (Fig. 2*A*, bottom panel). Accordingly, the thresholds for force initiation and cessation decreased as the X_m_ increased from minimal to maximal length (Fig. 2*B*). The capability of self-sustaining force generation (i.e., DCT) was increased by 52% at the optimal X_m_ (blue) and 90% at the maximal X_m_ (green) compared with the case without muscle spindle feedback (i.e., G_aff_ = 0; Fig. 2*B*,*C*, bottom panel). The rate of force development (i.e., 1/DCI) was 36% faster at the optimal X_m_ (blue) and 45% faster at the maximal X_m_ (green) in comparison to the case without muscle spindle feedback (i.e., G_aff_ = 0; Fig. 2*B*,*C*, bottom panel). The degree of force potentiation induced by motoneuron PIC activation (i.e., DFG) was increased by 140% for the optimal X_m_ (blue) and by 88% for the maximal X_m_ (green) relative to the case without muscle spindle feedback (i.e., G_aff_ = 0; Fig. 2*B*,*C*, bottom panel). The increase in DFG under muscle spindle feedback was attributable to the decrease in firing rate at the onset of firing acceleration induced by PIC activation in the motoneuron during the ascending phase. It is be notable that the force was potentiated by motoneuron PIC activation for all muscle lengths spanning the full physiological range. In summary, the increase in muscle length from physiological minimum to maximum could progressively lower the thresholds for initiation and cessation, increase the speed, and reduce the potentiation in force production during isometric contractions under somatic stimulation at the motoneuron.

### Influence of muscle spindle feedback on motor output under dendritic excitation

The effects of muscle length variation on motoneuron firing output and muscle force production were further investigated for synaptic drive over motoneuron dendrites (Fig. 3). Similar to the case of intracellular stimulation of the motoneuron at the soma (Fig. 2), the current thresholds for recruitment and derecruitment of the motoneuron during the triangular synaptic excitation (I_N_) were decreased by 35% and 78%, respectively, for the optimal X_m_ (blue) and by 71% and 183% for the maximal X_m_ (green) compared with the minimal X_m_ case (red; Fig. 3*A*,*C*, top panel). Accordingly, the force was initiated in the order of the maximal (green), optimal (blue), and minimal X_m_ (red; Fig. 3*B*). However, the capability of self-sustaining force generation (i.e., DCT), the rate of force development (i.e., 1/DCI) and the degree of force potentiation induced by motoneuron PIC activation (i.e., DFG) did not tend to be influenced by the muscle spindle feedback (i.e., G_aff_) during isometric contractions at different muscle lengths (Fig. 3*C*, bottom panel). This phenomenon occurred because the full activation of PICs over the motoneuron dendrites began at the initiation of firing under the synaptic drive to the motoneuron dendrites. In short, the increase in muscle length from physiological minimum to maximum could progressively lower the thresholds selectively for force initiation and cessation during isometric contractions under dendritic stimulation at the motoneuron.

### Interplay of PIC location and muscle length on motor output under somatic stimulation

**Figure 4.**
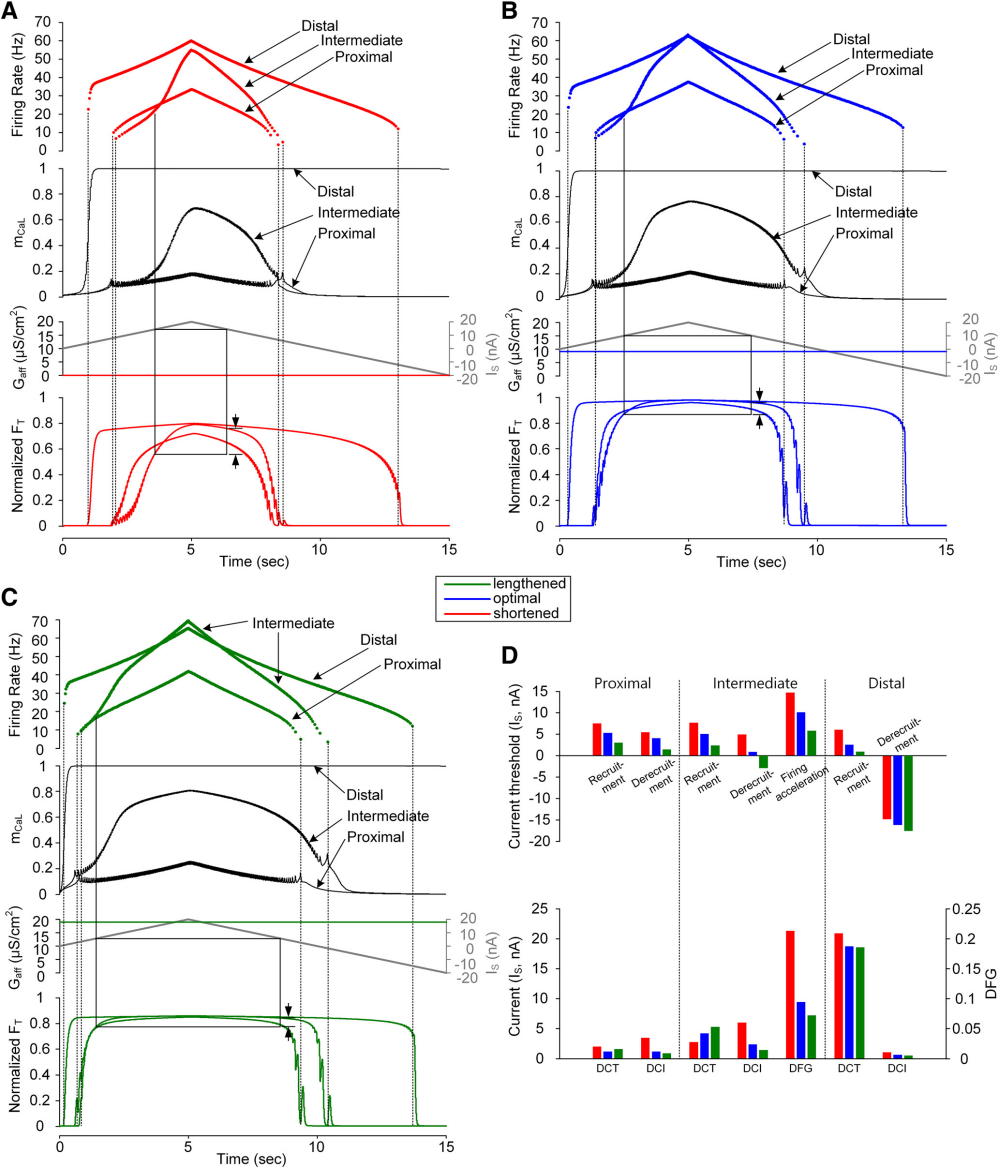
Somatic input-force output relationship of a model motor unit for variations in D_path_ and X_m_. Instantaneous firing rates of the motoneuron and average of activation gate dynamics for all L-type Ca_v_1.3 channels over the motoneuron dendrites (m_CaL_; top two panels), triangular current injected at the soma of the motoneuron (I_S_) and the afferent inputs from the muscle spindle (G_aff_; middle), and time courses of muscle force normalized with maximum force at the optical muscle length (bottom) under three types of PIC channel distribution [proximal (D_path_ = 0.2 mm), intermediate (D_path_ = 0.6 mm), and distal (D_path_ = 1.0 mm)] over the motoneuron dendrites during isometric contractions at shortened (X_m_ = −16 mm; ***A***), optimal (X_m_ = −8 mm; ***B***), and lengthened muscle length (X_m_ = 0; ***C***). Vertical dotted lines indicate the recruitment and derecruitment of the motor unit model. Vertical solid lines indicate the onset of firing acceleration by full PIC activation on the ascending stimulation phase and the same stimulation level for the firing acceleration on the descending stimulation phase. The gap between the two vertical arrows represents the degree of force potentiation induced by full PIC activation. ***D***, Bar graph for the current threshold for recruitment, derecruitment and PIC activation in the motoneuron (upper panel) and the characteristic indexes for motor output (bottom panel).

Having shown the influence of muscle length variation on motoneuron firing and muscle force, the location of PIC channels was varied among proximal (D_path_ = ∼0.2 mm) and distal (D_path_ = ∼1.0 mm) regions over the motoneuron dendrites. The three D_path_ conditions were selected for the dendritic sites over which the PIC channels were not fully activated, fully activated after firing onset, and fully activated at firing onset during the triangular current stimulation at the soma in the motoneuron, respectively. Figure 4 shows the dependence of PIC activation dynamics, motoneuron output and resulting force production on PIC location and muscle length during somatic stimulation of the motoneuron. Overall, the thresholds for recruitment (vertical dotted lines), firing acceleration (vertical solid lines), and derecruitment (vertical dotted lines) during triangular stimulation of the motoneuron at the soma (I_S_) decreased as the PIC location was moved farther from the soma (Fig. 4*A*, top panel). This result was attributed to the increases in dendritic input resistance (R_N,D_; Kim, 2017a, his Fig. 4) and PIC channel conductance (G_CaL_ in Table 1) over the distal regions of the dendrites. Accordingly, the initiation (vertical dotted lines) and cessation (vertical dotted lines) of muscle force occurred faster and slower, respectively, and the DCT increased as the D_path_ from the motoneuron soma increased (Fig. 4*A*,*D*, bottom panel). In contrast, the force potentiation (vertical arrows) induced by PIC activation (i.e., DFG) only occurred with intermediate D_path_ (i.e., ∼0.6 mm; Fig. 4*A*,*D*, bottom panel). This result could be explained by the lack of full PIC activation for PIC channels at the proximal location due to relatively low local R_N,D_ and G_CaL_ and high inhibition by AHP transduction and the simultaneous onset of PIC and firing for PIC channels at the distal location due to the relatively high local R_N,D_ and G_CaL_ and low inhibition by AHP transduction. Furthermore, the rate of force development (i.e., 1/DCI) was 71% faster for the proximal D_path_ and 447% faster for the distal D_path_ relative to those over the intermediate areas of the motoneuron dendrites (Fig. 4*A*,*D*, bottom panel). In other words, the discharge rate was lower for PIC channels in the intermediate location than for those in the proximal location before full PIC activation in the motoneuron, which is related to attenuation of the electrical signal flowing over passive dendrites (Rall and Rinzel, 1973; Zador et al., 1995; Kim et al., 2014). According to this cable theory, under somatic stimulation, the PIC channels located proximal to the soma would be more easily recruited and their currents more effectively transmitted to the soma than those located at the intermediate distance from the soma. For the PIC channels in the distal location, they began to fully activate at the time of motoneuron discharge due to high input resistance and large G_CaL_ over the distal dendritic sites.

The above results were consistent but of greater magnitude due to the increase in both afferent inputs and muscle activation while lengthening the muscle from the physiologically minimal to maximal length. The thresholds for force initiation (vertical dotted lines), potentiation (vertical solid lines) and cessation (vertical dotted lines) decreased with increasing muscle length for all three PIC locations (Fig. 4*B*,*C*). For the proximal D_path_, the thresholds for force initiation and cessation and their difference (i.e., DCT) were decreased by 30%, 25% and 41% at the optimal X_m_ (blue) and by 44%, 74% and 21% at the maximal X_m_ (green), respectively, compared with those at the minimal X_m_ (red; Fig. 4*D*). The decrease in the DCT with increasing X_m_ in the proximal D_path_ condition was attributed mainly to the decrease in recruitment threshold by the increase in G_aff_ and PIC activation level. For the intermediate D_path_, the thresholds for force initiation and cessation were decreased by 34% and 82% at the optimal X_m_ (blue) and by 52% and 159% at the maximal X_m_ (green), respectively, compared with those at the minimal X_m_ (red; Fig. 4*D*, top panel). However, the DCT was increased by 52% for the optimal X_m_ and 91% for the maximal X_m_ compared with those at the minimal X_m_ (Fig. 4*D*, bottom panel). The increase in DCT with increasing X_m_ in the intermediate D_path_ condition was caused mainly by the decrease in derecruitment threshold due to the increased G_aff_ and the full activation of PIC channels at higher current intensities than the threshold for firing initiation. For the distal D_path_, the thresholds for force initiation and cessation and the DCT were decreased by 58%, 9% and 10% at the optimal X_m_ (blue) and by 84%, 19% and 11% at the maximal X_m_ (green), respectively, compared with those at the minimal X_m_ (red; Fig. 4*D*). The decrease in DCT with increasing X_m_ in the distal D_path_ condition was derived mainly due to the decrease in recruitment threshold by the increase in G_aff_ and the full PIC activation at firing onset. In addition, the rate of force development (i.e., 1/DCI) with the proximal D_path_ was increased by 193% at the optimal X_m_ (blue) and by 286% at the maximal X_m_ (green) compared with that at the minimal X_m_ (red; Fig. 4*B–D*, bottom panel). For the intermediate D_path_ the rate of force development was increased by 147% at the optimal X_m_ (blue) and by 312% at the maximal X_m_ (green) compared with that at the minimal X_m_ (red; Fig. 4*B–D*, bottom panel). For the distal D_path_, the rate of force development was increased by 72% at the optimal X_m_ (blue) and by 100% at the maximal X_m_ (green) compared with that at the minimal X_m_ (red; Fig. 4*B–D*, bottom panel). Furthermore, under the intermediate D_path_ the threshold and degree (i.e., DFG) for force potentiation were decreased by 30% and 56% at the optimal X_m_ (blue) and by 62% and 66% at the maximal X_m_ (green) compared with that at the minimal X_m_ (red; Fig. 4*B*,*C*, vertical arrows, *D*).

In summary, as the distance to PIC channels from the cell body of the motoneuron increased the capability for self-sustaining force production was enhanced whereas the speed of force production was minimized and the force potentiation were maximized over the intermediate distances. In addition, the impact of muscle length variation on force production was also maximized when PIC channels were located over the dendritic sites separated by the intermediate distance from the soma under somatic stimulation of the motoneuron.

### Interplay of PIC location and muscle length on motor output under dendritic excitation

**Figure 5.**
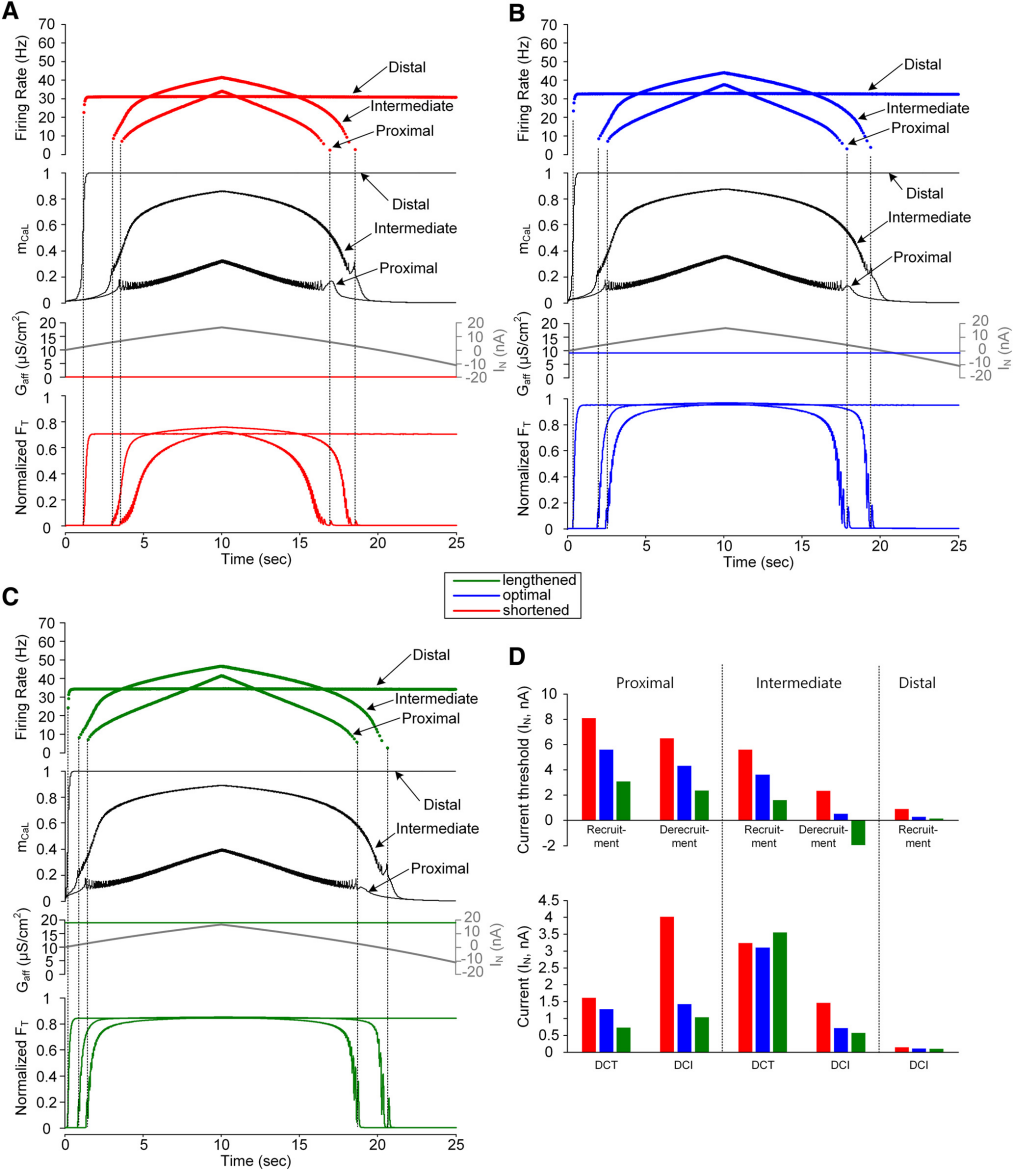
Dendritic input-force output relationship of a model motor unit for variations in D_path_ and X_m_. Instantaneous firing rates of the motoneuron and average of activation gate dynamics for all L-type Ca_v_1.3 channels over the motoneuron dendrites (m_CaL_; top two panels), effective synaptic current approaching the soma during the triangular variation in the peak conductance of the synaptic inputs over the passive dendrites of the motoneuron (I_N_) and the afferent inputs from the muscle spindle (G_aff_; middle), and time courses of muscle force normalized with maximum force at the optical muscle length (bottom) under three types of PIC channel distribution [proximal (D_path_ = 0.2 mm), intermediate (D_path_ = 0.6 mm), and distal (D_path_ = 0.1 mm)] over the motoneuron dendrites during isometric contractions at shortened (X_m_ = −16 mm; ***A***), optimal (X_m_ = −8 mm; ***B***), and lengthened muscle length (X_m_ = −16 mm; ***C***). Vertical dotted lines indicate the recruitment and derecruitment of the motor unit model. ***D***, Bar graph for the current threshold for recruitment and derecruitment in the motoneuron (upper panel) and the characteristic indexes for motor output (bottom panel). Note the lack of full PIC activation for proximal PIC location and the simultaneous onset of firing and PIC for intermediate and distal PIC location during the ascending stimulation phase.

The interaction of PIC location and muscle length on PIC activation dynamics, motoneuron output and force generation was further investigated for dendritic stimulation of the motoneuron (Fig. 5). As shown in the somatic stimulation case (Fig. 4), the thresholds (vertical dotted lines) for force initiation and cessation decreased but the DCT increased with increasing D_path_ to the PIC channels on the motoneuron dendrites (Fig. 5*A*,*D*, bottom panel). However, the force potentiation (i.e., DFG) was not obvious due to the lack of full PIC activation for proximal D_path_ and the simultaneous onset of PIC and firing in the motoneuron for intermediate and distal D_path_ during the ascending stimulation phase (Fig. 5*A*). In addition, the rate of force development (i.e., 1/DCI) gradually increased as the PIC channels were located farther from the cell body in the motoneuron (Fig. 5*A*,*D*, bottom panel). It is notable that the saturation and reduction of firing rate for distal PIC channels in the motoneuron were attributed to the large peak conductance of PIC channels (G_CaL_ in Table 1) along with high input resistance over distal dendritic branches (R_N,D_; Kim, 2017a, his Fig. 4) and the low signal transduction from the distal dendrites to the soma (Kim et al., 2014, their Fig. 1).

These results were also consistent but of greater magnitude due to the increase in both afferent inputs and muscle activation as the muscle length was varied from the physiologically minimal to maximal length. The thresholds for force initiation and cessation decreased with increasing muscle length for all three PIC locations (Fig. 5*B*,*C*, vertical dotted lines). For the proximal D_path_, the thresholds for force initiation and cessation and their difference (i.e., DCT) were decreased by 31%, 33% and 21% at the optimal X_m_ (blue) and by 62%, 64% and 55% at the maximal X_m_ (green) compared with the minimal X_m_ (red; Fig. 5*D*). The decrease in the DCT with increasing X_m_ in the proximal PIC condition was attributed mainly to the decrease in recruitment threshold by the increase in G_aff_ and PIC activation level. For the intermediate D_path_, the thresholds for force initiation and cessation were decreased by 35% and 78% at the optimal X_m_ (blue) and by 71% and 183% at the maximal X_m_ (green) compared with the minimal X_m_ (red; Fig. 5*D*, top panel). However, the DCT did not tend to be influenced by increase in X_m_ due to the simultaneous onset of firing initiation and full PIC activation in the motoneuron (Fig. 5*B*,*C*,*D*, bottom panel). For the distal D_path_, the threshold for force initiation was decreased by 70% at the optimal X_m_ (blue) and by 86% at the maximal X_m_ (green) compared with the minimal X_m_ (red; Fig. 5*D*, top panel). Under the distal D_path_, the force did not cease at the end of synaptic stimulation of the motoneuron dendrites for any of the three muscle lengths (Fig. 5*A–C*). Furthermore, the rate of force development (i.e., 1/DCI) for the proximal D_path_ was increased by 180% at the optimal X_m_ (blue) and by 288% at the maximal X_m_ (green) compared with the minimal X_m_ (red; Fig. 5*D*, bottom panel). For the intermediate D_path_, the rate of force development was increased by 106% at the optimal X_m_ (blue) and by 156% at the maximal X_m_ (green) compared with the minimal X_m_ (red; Fig. 5*D*, bottom panel). For the distal D_path_, the rate of force development was increased by 33% at the optimal X_m_ (blue) and by 49% at the maximal X_m_ (green) compared with the minimal X_m_ (red; Fig. 5*D*, bottom panel).

To this end, under dendritic stimulation of the motoneuron, the capability for self-sustaining force production was enhanced with increasing distance to PIC channels from the cell body of the motoneuron. Unlike the case of somatic stimulation, however, the speed of force production was gradually increased and the force potentiation did not occur at all three distances. In addition, the impact of muscle length variation on the rate of force development was maximized when Ca_v_1.3 channels were located proximally to the soma.

### Link between PIC location-muscle length interaction and motor function under somatic stimulation

**Figure 6.**
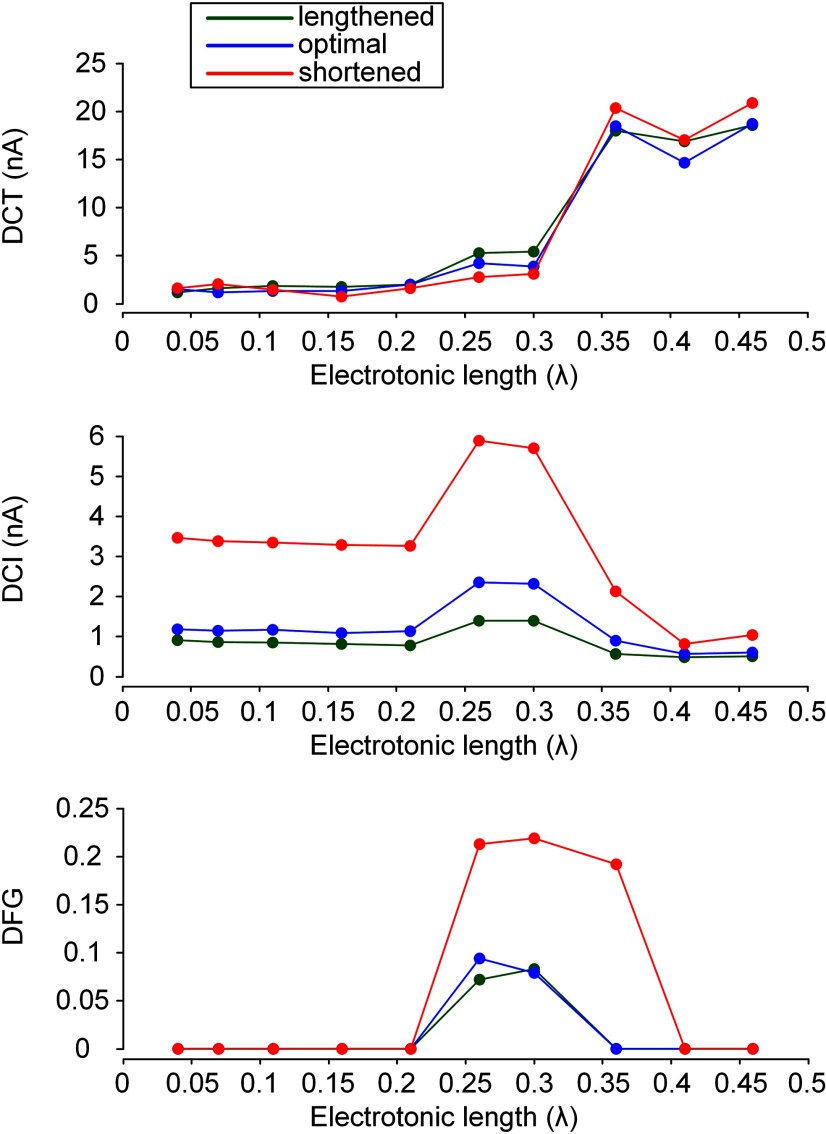
Somatic input-force output relationship of a model motor unit over a wide range of PIC locations. The three characteristic indices (DCT, DCI, and DFG) were calculated from the input-output curve of the model motor unit while varying the location of PIC channels over the motoneuron dendrites during isometric contractions at shortened (X_m_ = −16 mm), optimal (X_m_ = −8 mm), and lengthened muscle length (X_m_ = 0 mm). The same stimulation protocol used in Figure 2 was applied to the model motoneuron. The three characteristic indices were plotted against the mean of electrotonic lengths (λ) to individual dendritic sites where the PIC channels were located. Filled circles indicate ten samples of PIC channel locations ranging from the D_path_ of 0.1 to 1.0 mm with the interval of 0.1 mm (Table 1).

The results from simulations of the closed-loop motor unit model for the three samples of PIC locations over the motoneuron dendrites were validated over a broad range of the mean of electrotonic lengths calculated at individual dendritic sites where the PIC channels were located away from the soma by D_path_ along the motoneuron (Table 1). Figure 6 shows the overall trends of the variations in the capability of self-sustaining force production (i.e., DCT), the rate of force development (i.e., 1/DCI) and the degree of force potentiation by motoneuron PIC activation (i.e., DFG) for three (physiologically minimal, optimal and maximal) muscle lengths under triangular current stimulation at the soma. As predicted in Figure 4, the self-sustaining force production capability increased as the D_path_ (or electrotonic length) increased from the soma of the motoneuron. The variability in the DCT was due to the variation in afferent inputs from the muscle spindle at different muscle lengths, which indicated much less effect of muscle length on DCT than PIC location. The rate of force development was minimized over the intermediate range (0.5–0.8 mm or 0.21λ–0.36λ) of D_path_ (or electrotonic length) for all three muscle lengths. The degree of force potentiation was also prominent over the intermediate range (0.5–0.8 mm or 0.21λ–0.36λ) of D_path_ (or electrotonic length). It is notable that the rate and potentiation of force production were influenced to an even greater degree by the variation in PIC location for shorter-than-optimal muscle lengths than for longer-than-optimal muscle lengths. These results suggest that under the somatic stimulation of the motoneuron the minimal speed and maximal potentiation of force production would occur with the PIC channels located over the intermediate distances from the soma of the motoneuron and the muscle length shortened below the optimal length.

### Link between PIC location-muscle length interaction and motor function under dendritic excitation

**Figure 7.**
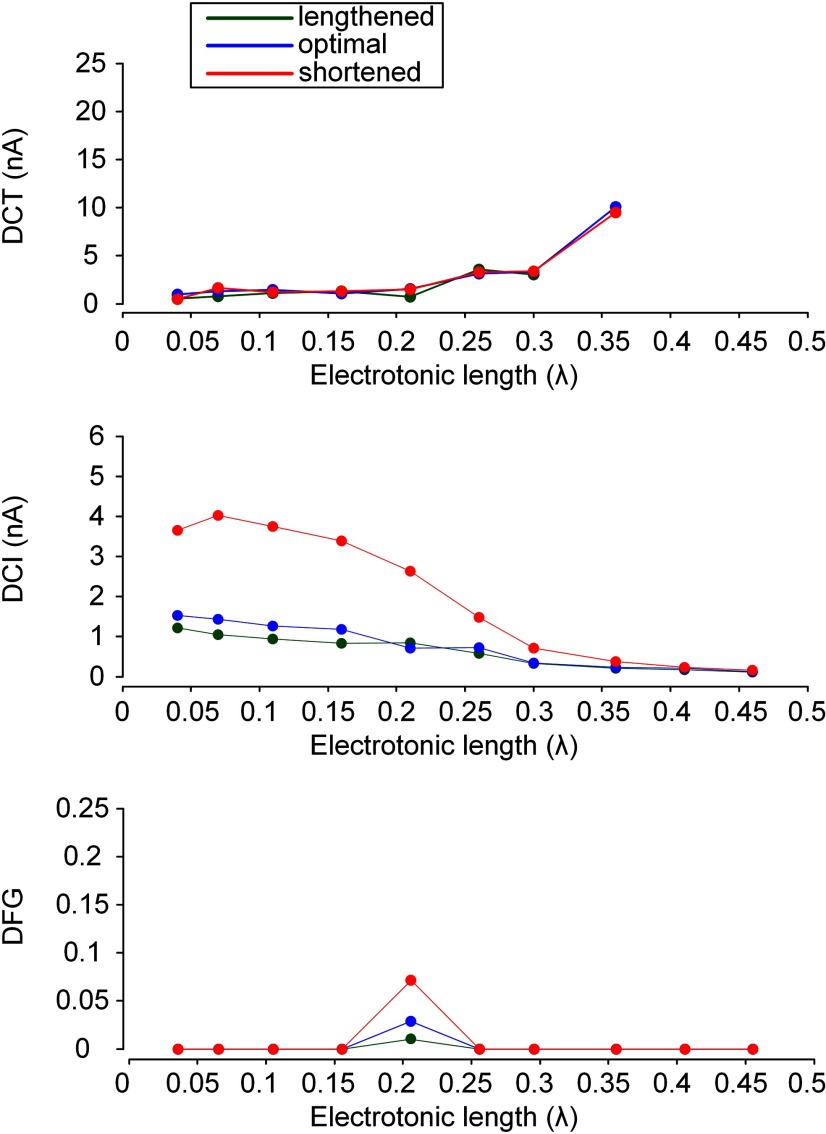
Dendritic input-force output relationship of a model motor unit over a wide range of PIC locations. The three characteristic indices (DCT, DCI, and DFG) were operationally calculated from the input-output curve of the model motor unit while varying the location of PIC channels over the motoneuron dendrites during isometric contractions at shortened (X_m_ = −16 mm), optimal (X_m_ = −8 mm), and lengthened muscle length (X_m_ = 0 mm). The same stimulation protocol used in Figure 3 was applied to the model motoneuron. The three characteristic indices were plotted against the mean of electrotonic lengths (λ) to individual dendritic sites where the PIC channels were located. Filled circles indicate ten samples of PIC channel locations ranging from the D_path_ of 0.1 to 1.0 mm with the interval of 0.1 mm (Table 1).

Similar results were found under the dendritic excitation condition of the motoneuron. Figure 7 shows the changes in the capability of self-sustaining force production (i.e., DCT), the rate of force development (i.e., 1/DCI) and the degree of force potentiation (i.e., DFG) resulting from the variation in motoneuron PIC location in muscles with decreased, optimal and increased lengths for triangular excitatory synaptic inputs to the motoneuron dendrites. The capability of self-sustaining force production was increased with increasing path length (or electrotonic length) to the PIC channels. Unlike the somatic stimulation case, the rate of force development gradually increased as the D_path_ (or electrotonic length) increased for all three muscle lengths. Force potentiation was reduced and observed only in a limited range of D_path_ (or electrotonic length) values around 0.5 mm (or 0.21λ) during isometric muscle contraction for all three muscle lengths. These differences under the dendritic stimulation condition resulted from the direct excitation of PIC channels by the synaptic inputs placed over the motoneuron dendrites. It is notable that under dendritic excitation of the motoneuron the influence of PIC location on the rate of force production was more prominent for shorter-than-optimal muscle lengths than for longer-than-optimal muscle lengths. Based on these results, it might be predicted that under the dendritic excitation of the motoneuron the speed of force generation would be minimized with the PIC channels proximal to the soma of the motoneuron and the muscle length shortened below the optimal length. In addition, the force potentiation might be maximized with the PIC channels located over the intermediate distances from the soma of the motoneuron at the optimal muscle length. For both somatic and dendritic stimulation in the motoneuron, the impact of muscle shortening on the speed of force production would be expected to be stronger with the PIC channels proximal than distal to the cell body of the motoneuron.

### Interplay of PIC location and muscle length on warm-up behavior under somatic stimulation

**Figure 8.**
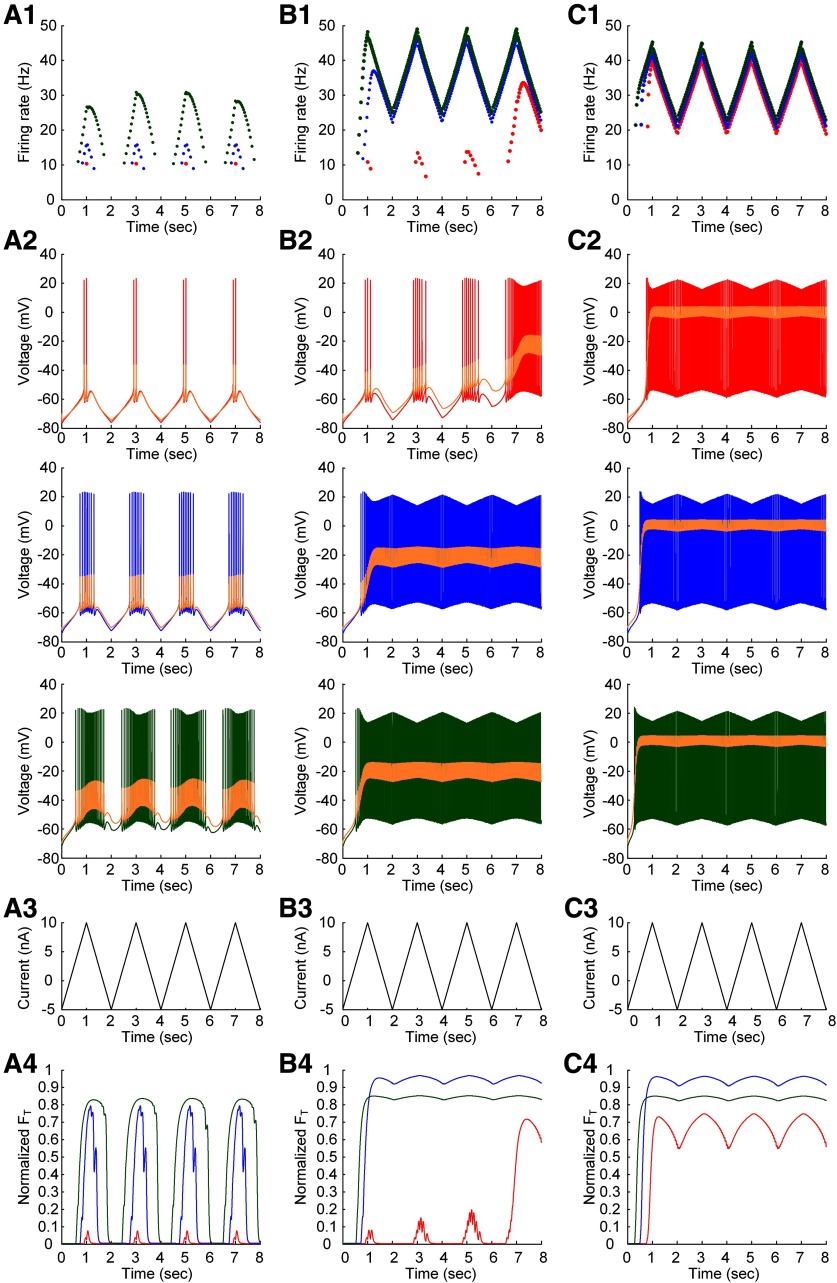
Warm-up behavior of a model motor unit under the somatic stimulation. The simulations were conducted for three PIC locations [D_path_ = 0.6 mm (***A***), 0.8 mm (***B***), and 1.0 mm (***C***)] during isometric contractions at the three muscle lengths [X_m_ = −16 mm (red), −8 mm (blue), and 0 mm (green)]. ***A1***, ***B1***, ***C1***, Instantaneous firing rates at the three muscle lengths. ***A2***, ***B2***, ***C2***, Membrane potentials of the motoneuron at the three muscle lengths. Note that the average of membrane potentials measured over the dendritic sites for PIC channels was plotted in orange color. ***A3***, ***B3***, ***C3***, Current stimulation injected at the soma (solid black) and muscle afferent inputs over the dendrites in the motoneuron at the three muscle lengths. ***A4***, ***B4***, ***C4***, Isometric muscle forces at the three muscle lengths. All muscle forces were normalized with the peak force measured at the optimal muscle length. Note the gradual increase in firing rate of the motoneuron and force production of the muscle in ***B1***, ***B4*** with the repeated stimulation at the soma of the motoneuron during the isometric contraction at the shortened muscle length.

Lastly, it was evaluated how the interplay of PIC location and muscle length might influence the gradual increase of response (or warm-up) with repeated activation in the closed-loop motor unit. Figure 8 shows the simulation results obtained during the repetition of triangular current stimulation at the soma of the motoneuron. As shown in the previous study (Bennett et al., 1998), the motoneuron could demonstrate the gradual decrease in thresholds for firing initiation and termination and the gradual increase in hysteresis (difference between current thresholds for firing initiation and termination) with repeated somatic stimulation (Figure 8*B1*,*B2*, red dots and lines, and compare with Bennett et al., 1998, their Fig. 3). Accordingly, the motoneuron and muscle unit could display the progressive increase in firing rate and force production with the PIC channels located at the D_path_ (or electrotonic length) of 0.8 mm (or 0.36λ) from the soma during isometric contractions at the shortened X_m_ (−16 mm; Fig. 8*B4*, red lines). This warm-up firing and resulting force potentiation were contributed to the residual activation of PIC channels due to their slow kinetics over the motoneuron dendrites during the repeated stimulation at the soma of the motoneuron (Fig. 8*B2*, orange line in the top panel). However, the warm-up behavior and force potentiation tended to disappear either when the PIC channels were moved away from the D_path_ of 0.8 mm (Fig. 8*A*,*C*) or when the X_m_ was lengthened beyond the optimal length (−8 mm; Fig. 8*B*, blue and green lines). The absence of warm-up behavior was mainly due to the relatively low local R_N,D_ and G_CaL_ at D_path_ of 0.6 mm (or 0.26λ) and the relatively high local R_N,D_ and G_CaL_ at D_path_ of 1.0 mm (or 0.46λ) in comparison to the case at D_path_ of 0.8 mm (or 0.36λ). In addition, the warm-up behavior was not apparent for muscles longer than the optimal X_m_ at the D_path_ of 0.8 mm (or 0.36λ) because of the excitatory afferent inputs from the muscle to the motoneuron dendrites. Consistent with previous simulation results, the force potentiation induced by the warm-up behavior of the motoneuron with the repeated stimulation was apparent with the PIC channels located over the intermediate distances from the soma and the muscle length shortened below the optimal length. These results indicate that the warm-up behavior of the motor unit system depends on the location of PIC activation over motoneuron dendrites and the state of muscle length.

### Interplay of PIC location and muscle length on warm-up behavior under dendritic excitation

**Figure 9.**
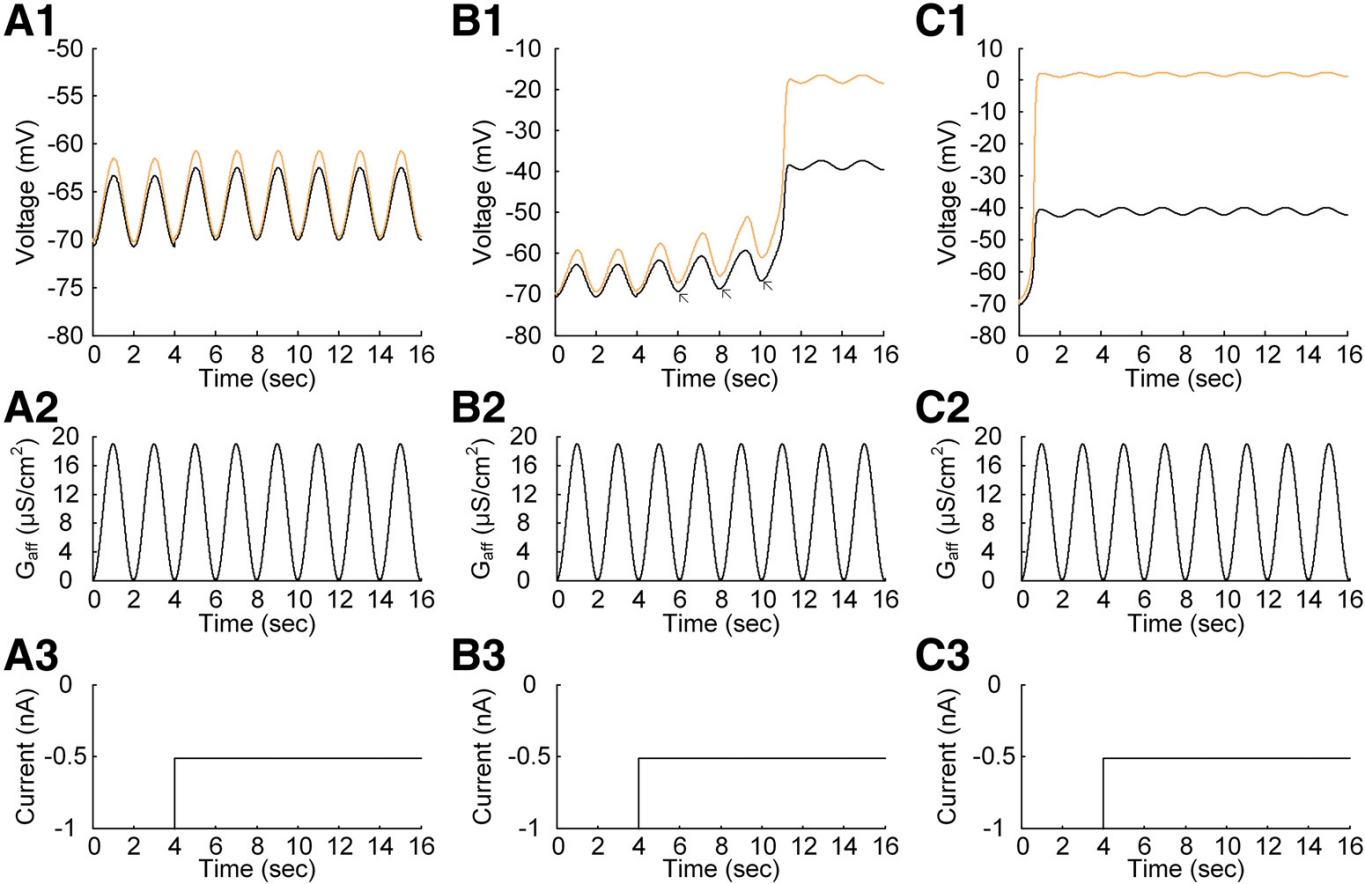
Warm-up behavior of a model motor unit under the dendritic excitation. The simulations were conducted for three PIC locations [D_path_ = 0.6 mm (***A***), 0.8 mm (***B***), and 1.0 mm (***C***)] while varying the maximum conductance of muscle afferent inputs over the motoneuron dendrites in a sinusoidal form. Note that the minimum and maximum value of G_aff_ correspond to those estimated under the physiologically minimal (X_m_ = −16 mm) and maximal (X_m_ = 0 mm) muscle length, respectively (for details, see the Materials and Methods). ***A1***, ***B1***, ***C1***, Membrane potential at the soma (black) and the average of membrane potentials measured over the dendritic sites for PIC channels (orange). ***A2***, ***B2***, ***C2***, Sinusoidal variation in the maximum conductance (G_aff_) of muscle afferent inputs over the motoneuron dendrites. ***A3***, ***B3***, ***C3***, Step current injected at the soma of the motoneuron. Note the gradual increase (indicated by arrows) in membrane potentials at the soma and the dendritic region for PIC channels with the repeat activation of muscle spindle afferents.

The current model of closed-loop motor unit system with muscle spindle feedback could also replicate the warm-up behavior of the motoneuron that has been observed under dendritic excitation during sinusoidal tendon movement from physiologically minimal and maximal muscle length over a 2-s period in the previous experimental study (Bennett et al., 1998). The inputs from this tendon vibration to the motoneuron were simulated by varying the maximum conductance (G_aff_) of afferent synapses over the motoneuron dendrites in a sinusoidal form with the period of 2 s. Figure 9 shows the interplay of PIC location and muscle length on the warm-up phenomenon under the sinusoidal tendon vibration protocol. The motoneuron model could accurately reproduce the experimental observations of gradual increase and delayed plateau in membrane potential with repeated stimulation over the dendrites (compare Bennett et al., 1998, their Figs. 9*B1* and 4). Similar to the case of somatic stimulation, the warm-up phenomenon of the motoneuron with the repeated tendon movement was apparent with the PIC channels located over the intermediate distances from the soma (i.e., D_path_ = 0.8 mm or 0.36λ) and the muscle length shortened below the optimal length (i.e., X_m_ < −8 mm). This warm-up behavior was attributed to the residual activation of PIC channels due to their slow kinetics over the motoneuron dendrites during the repetition of tendon movement in a sinusoidal form (Fig. 9*B1*, orange line). However, the warm-up behavior disappeared when the PIC channels were moved away from the D_path_ of 0.8 mm (or 0.36λ; Fig. 9*A*,*C*). This result was consistent with the result under somatic stimulation and attributable to the lower R_N,D_ and G_CaL_ at D_path_ of 0.6 mm (or 0.26λ) and the higher R_N,D_ and G_CaL_ at D_path_ of 1.0 mm (or 0.46λ) relative to the case for D_path_ of 0.8 mm (or 0.36λ).

## Discussion

Using a physiologically realistic model of the closed-loop motor unit, it has been demonstrated that the interplay of PIC activation location and muscle fascicle length may play an important role in shaping motor output at the single motor unit level during motor activities. The simulation results of the present study systematically revealed a functional link between PIC location and motor performance by the closed-loop motor unit for control of movement. The results from the simulations may provide comprehensive insights into how central (motoneuron PIC) and peripheral (muscle length) factor interact to shape the motor output under isometric muscle activities at various levels of muscle length in the motor unit system. Thus, these insights may be useful not only for interpretation of physiological measures but also for designing novel experiments and data analysis on motoneuron and motor unit behaviors during motor activities.

### Functional role of muscle spindle afferents in motoneuron firing and force output

To our knowledge, few studies have been quantitatively conducted on the motor consequences of afferent inputs over motoneuron dendrites at the motor unit level under various muscle length states. In the present study, the synaptic inputs from the muscle spindle were realistically distributed over the motoneuron dendrites including PIC channels up to D_path_ values of 1.4 mm based on the previous study on the spatial distribution of group Ia afferent inputs over motoneuron dendrites (Segev et al., 1990). The conductance of the afferent inputs was also physiologically determined based on voltage clamp data obtained from adult cat motoneurons under passive conditions while slowly varying the muscle length (Lee et al., 2003). As the level of afferent inputs increased with increasing muscle lengths from physiologically minimum to maximum values, the threshold for firing initiation was monotonically reduced ∼fourfold at the physiologically maximal length under both somatic and dendritic stimulation condition (Figs. 2*A*, 3*A*). Consequently, the firing rate at the firing threshold for the physiologically minimal muscle length could be monotonically increased by almost fourfold when the muscle length was varied to the physiological maximum. Likewise, the force initiation and magnitude at the physiologically minimal length became gradually faster up to almost threefold and larger up to almost peak force as the muscle length increased to the physiological maximum (Figs. 2*B*, 3*B*). Furthermore, the speed of force development (i.e., 1/DCI) and the degree of force potentiation (i.e., DFG) could also be gradually enhanced under the somatic stimulation condition (Fig. 2*C*). In addition, the transient inhibition of ankle extensor motoneurons by Ib afferents observed during isometric contractions of triceps surae is unlikely to influence the slow dynamics of PIC activation due to the quick decrease of Ib inhibition probably by presynaptic inhibition at Ib terminals in cats (Zytnicki et al., 1990; Lafleur et al., 1992). In fact, this inhibitory coupling between muscle contraction and afferent feedback was not sufficient to prevent the force potentiation probably induced by the activation of PICs over motoneuron dendrites during electrical stimulation of triceps surae under isometric condition in humans (Collins et al., 2001, 2002). All these results emphasize that the functional influence of the feedback signals from the muscle spindle on motoneuron discharge and muscle force should be considered for correct interpretation of motor unit data. In the physiological perspective of motor control, the systematic interplay of muscle spindle feedback, PIC channels and excitation condition in the motoneuron may be beneficial to precisely control the force output of the motor unit not only for improving motor performance but also for preventing muscle damage during lengthening contractions by limiting the amount of muscle lengthening.

### Potential impact of PIC activation location on motor output during movement

Three out of the four patterns of input-output relationship experimentally identified in rodent motoneurons under somatic stimulation (Bennett et al., 2001) could be reproduced by varying the PIC channel location on motoneuron dendrites. When the PIC channels were placed at dendritic regions proximal to the soma, the motoneuron could display the linear input-output relationship without nonlinear features such as frequency acceleration and self-sustaining firing during triangular stimulation of the soma (i.e., Type I). For PIC channels located at intermediate dendritic regions, firing acceleration occurred at higher current than that for firing initiation during the ascending phase, and self-sustaining firing was clearly observed during the descending phase below the current threshold for firing initiation, showing counterclockwise hysteresis (i.e., Type IV). For PIC channels located at dendritic regions distal to the soma, firing acceleration tended to occur simultaneously with firing initiation, and the capability of self-sustaining firing was greatly expanded (i.e., Type III). This location dependency describing the mode of PIC channel activation can be attributed in part to the nonuniform distribution of input resistance and direction-dependent signal propagation over the motoneuron dendrites (Zador et al., 1995; Kim et al., 2014). In accordance, at the motor unit level, the thresholds for force initiation, potentiation and cessation were decreased, and the rate of force development was increased with increasing path length to the location of Ca_v_1.3 channels on the motoneuron dendrites (Figs. 4, 5).

However, the locational effects of PIC activation on motoneuron output were not equally reflected in the motor output but selectively depended on the mode of motor activity. In fact, the changes in motoneuron firing by the variation in PIC location could more strongly affect force output during muscle contractions at shorter-than-optimal lengths (see DCI and DFG; Figs. 6, 7). This result was related to the degradation of muscle activation that has been observed for shorter-than-optimal muscles under low frequency (i.e., <20 Hz) motoneuron firing (Perreault et al., 2003; Kim et al., 2015). In particular, the rate of force development and the degree of force potentiation were not highly influenced by variations in PIC location for muscles with longer-than-optimal lengths. This result is due to the fact that muscle activation reached almost the peak level for longer-than-optimal muscle lengths, under which the muscle force did not tend to be greatly influenced by the variation in motoneuron firing due to changes in PIC location on the motoneuron dendrites.

Based on these simulation results, several insights can be derived for the interpretation of physiological measurements from human studies. First, the variability in voluntary force production during isometric contractions has been reported, and possible mechanisms have been suggested, including synaptic noise by bombardment of background signals (Calvin and Stevens, 1968; Jones et al., 2002), oscillations in descending motor commands (Watanabe and Kohn, 2015; Lodha and Christou, 2017), orderly recruitment of motor units with different twitch amplitudes (Jones et al., 2002), and delayed feedback signals from the muscle spindle and Golgi tendon organ (Lippold, 1970; Stein and Oguztorel, 1976; Christakos et al., 2006). The simulation results from the present study suggest that the variability in the threshold for motor unit recruitment and derecruitment induced by the variability in the location of dendritic PIC channels among spinal motoneurons may exacerbate the force variability as a cellular mechanism. For instance, the threshold change of force initiation by variation in PIC location may cause phase shift in force wave of individual motor units. The variability of force output in motor unit pool, thus, may be facilitated in in-phase condition between force waves that single motor units produce. Second, the neural excitation of muscles has been shown to be modulated more strongly for shortening contractions than for lengthening contractions (Petersen et al., 2007; Duchateau and Enoka, 2008). The intervention of motoneurons by motor commands from the brain might differ not only due to differences in muscle activation level but also to differences in the impact of PIC location on force production between shortening and lengthening contractions (central mechanism). Third, the cellular mechanism underlying spasm or dystonia after spinal cord injury has been suggested to involve malfunctioning of PIC activation in motoneuron dendrites (Li et al., 2004; Murray et al., 2010). Changes in PIC location along motoneuron dendrites during this pathologic condition might be a factor causing movement disorders (cellular mechanism). Lastly, mouse motoneurons have also been demonstrated to pertain PICs (2.39 ± 1.96 nA) responsible for bistable behavior that are not significantly smaller in comparison with cats (3.38 ± 4.47 nA; Manuel et al., 2019). However, mouse motor units have been reported to produce almost all force (∼90%) on the onset of steady regular firing (30–70 Hz; Manuel and Heckman, 2011). Thus, the influence of motoneuron PIC location and muscle length variation on the rate of force development (1/DCI) and the degree of force potentiation (DFG) shown in Figures 6, 7 may be considerably low for small species of which motoneurons have typically small dendrites and high excitability.

### Functional consequences of neuromodulatory versus PIC activation location

Recent experimental studies have suggested that the level of neuromodulatory drive from the brain stem may vary depending on the target muscles (Bowker et al., 1982; Powers and Binder, 2001; Esposito et al., 2014) and pathologic states (McPherson et al., 2018a,b). A recent computational study showed analyzing the open-loop motor unit model how neuromodulatory inputs to motoneurons, including intrinsic inputs from PIC channels at a D_path_ of ∼0.6 mm, influence force production by the motor unit during isometric contractions at various muscle lengths (Kim, 2017b). Under dendritic excitation condition, the capability of self-sustaining firing (i.e., DCT) and the rate of force development (i.e., 1/DCI) increased with increasing neuromodulatory input from −30% to 40% deviation from the default level (Kim, 2017b, his Fig. 8) and the neuromodulation effect on the 1/DCI was more obvious for shortened muscles. These results from the previous study were qualitatively similar to those in the present study, in which PIC channels were moved eccentrically away from the soma (i.e., D_path_ of 0 to ∼1 mm; compare Kim, 2017b, his Figs. 7 and 8). However, the discharge patterns of the motoneuron and the degree of force potentiation (i.e., DFG) considerably differed between two cases. For the dendritic excitation, the peak firing rate progressively increased with increasing neuromodulatory drive whereas the peak firing rate was lower for PIC location distal than intermediate to the soma at all muscle lengths (Fig. 5). The PIC-induced force potentiation was found over a very limited range of D_path_ (i.e., ∼0.5 mm or 0.21λ) to PIC channels over the motoneuron dendrites (Fig. 7). To this end, locational variation in PIC activation over motoneuron dendrites may lead to the distinctive effects of variation in the level of neuromodulatory drive on the discharge patterns of motor units and the time courses of force development in skeletal muscles. The combinational influence of neuromodulation and PIC location might enrich the computational capability of spinal motoneurons in controlling muscle force for proper movements.

### Robustness of force output under variations in PIC location and muscle length

Despite many research attempts, how PIC channels are distributed over motoneuron dendrites remains uncertain (Heckman and Enoka, 2012). One computational study suggested a linear relationship between PIC location and dendrite size (Grande et al., 2007). Another study showed that PIC location may be related to the electrical properties of motoneurons rather than dendritic size (Kim, 2017a). Furthermore, the electrical structure of dendrites has been demonstrated to be a function of dendritic morphology (Mainen and Sejnowski, 1996; Magee, 2000) and passive membrane properties (Kim et al., 2009; Kim and Jones, 2012). Thus, the effects of PIC location on the motor output of motor units might vary with changes in the morphologic and electrical factors of dendrites during normal development (Miller, 1981; Cline, 2001) or with pathologic damage (Elbasiouny et al., 2010; Šišková et al., 2014). However, the results of the present study indicated that the impact of PIC location variation on motor output might be mostly prominent when the muscle is shortened below its optimal length. Thus, it is proposed that the closed-loop motor unit system might be designed to maintain motor performance (i.e., rate and amplitude of force development) more robustly for isometric contractions at optimal and longer lengths under the circumstance that the location of PIC channels along spinal motoneurons could have some variance. In addition, the larger degree of force potentiation for shortened muscles might play a role as a compensatory mechanism for the degradation of muscle activation during shortening muscle contractions.

### Modeling considerations

The open-loop motor unit model developed in the previous study was extended for the present study, including the physiological distribution of afferent inputs from the muscle spindle over the motoneuron dendrites. The fundamental limitations of the current modeling approach have been discussed in the previous study (Kim, 2017b). For further investigation of the closed-loop motor unit system under physiological intrinsic and extrinsic conditions, the motor unit model presented here should be improved considering the following issues. First, the PIC-generating mechanisms need to be specified to produce realistic shapes of plateau potentials and PIC currents measured at the soma by including other types of channels, such as calcium-dependent potassium channels and persistent sodium channels, and the dynamics of the calcium reversal potential (Powers et al., 2012). Second, the type-specific electrical properties, such as input resistance, membrane time constant, rheobase current and AHP, observed in other types of motor units might lead to type-related influence of PIC activation on motor output of the motor unit (Zengel et al., 1985). Furthermore, future investigations would be needed for the interplay of PIC location and muscle length on motor function in small animals that include small motoneuron dendrites (Amendola and Durand, 2008) and more excitable membrane properties (Manuel et al., 2009). Third, the mechanical coupling between motor unit contraction and muscle spindle feedback should be taken into account depending on the type of muscle (Brizzi et al., 2002) and the anatomic arrangement between the motor unit and spindle receptor (Binder and Stuart, 1980). Finally, synaptic background noise and inhibitory synaptic inputs could be involved and alter the input-output properties of the motor unit during joint movements (Fellous et al., 2003; Hyngstrom et al., 2007).

## Conclusions

The location of PIC activation over motoneuron dendrites may be functionally linked to the characteristics of motor output during motor activities. In particular, the motor performance during shortening muscle contraction may be distinctively altered in accordance to the change in PIC location over motoneuron dendrites. This result may provide a basis for evaluating the location of PIC activation over spinal motoneurons based on characterization of motor outputs. The present study would be useful to investigators interested in spinal mechanisms and motor unit function in control of movement.

## Synthesis

Reviewing Editor: Dritan Agalliu, Columbia University Medical Center Decisions are customarily a result of the Reviewing Editor and the peer reviewers coming together and discussing their recommendations until a consensus is reached. When revisions are invited, a fact-based synthesis statement explaining their decision and outlining what is needed to prepare a revision will be listed below. The following reviewer(s) agreed to reveal their identity: NONE.

Authors have investigated the role of location PIC (persistent inward current) channel and muscle length on motor performance in a closed loop motor unit (with f/b from muscle spindle). Their simulations show faster force development and its sustenance when PIC is on Soma or dendrite, but that varied with muscle length. Their simulations, if valid, lay out some interesting properties related to muscle dynamics. The simulations show faster force development and its sustenance when PIC is on soma or dendrite, but this varied with muscle length. Although single neuron modeling is not my area, the results seem novel. Paper is well written.

There are some minor concerns for the study:

1. I would like authors to add a bit more to the discussion on how muscle length variations during real world situations (physiological minimum and maximum lengths) alter threshold for force initiation during contraction.

2. Fig 2c needs to have error bars.

3. The physiological relevance is not that clear to me. The authors need to explain that better in the discussion.

4. The significance statement should provide a better explanation of the relevance of the research and the importance in the field.
